# The Problem of the Poor in London

**Published:** 1894-11-10

**Authors:** 


### THE PROBLEM OF THE POOR IN LONDON.

Sir G. R. Tyler, Bart., Lord Mayor, writes from the Mansion House, London: Will you allow me to appeal through your columns for a measure of personal service on the part of a few residents in or near London in connection with this subject? Early in last year a conference of the titular heads of the Churches of all denominations, and other persons, was convened by my predecessor to consider the question of dealing with London poverty. The matter was referred to a committee, who, after much inquiry and deliberation, proposed the establishment of friendly workers' areas, under the management of inter-denominational committees, whereby the inhabitants of each district of London could take charge of their own poor, with every hope of effectually grappling with the problem presented in those centres where the scheme was put into force. At a further meeting of the conference in December, 1893, it was determined to empower the committee to select four experimental areas in London to test the practicability of the proposed scheme. Since then the committee have conferred with the local clergy and residents in certain selected areas, with the result that arrangements have been made to commence the work so soon as a few laymen of earnest purpose can be found to accept office as chairmen and honorary secretaries of the inter-denominational committees The selected areas fairly represent all types of the population, and the position of chairman of a committee would open up a wide field of interesting work for any person of administrative capacity with reasonable leisure. London is so vast and the selected districts are relatively so poor, that the committee have no means of finding the necessary officers to make the scheme a success, except through the Press. I have, therefore, pleasure in inviting persons of experience and leisure, who are willing to fill the posts of chairmen and honorary secretaries of the inter-denominational committees now in course of organization, to communicate with me at the Mansion House. I may add that the services of young men to undertake the duties of honorary secretaries are desired equally with those of men of maturer years to fill the more responsible posts of chairmen. The inter-denominational committee is an interesting experiment, which, if successful (as with sufficient voluntary personal service it ought to be), will inaugurate a work likely to confer great and far-reaching benefits upon the poor of London and its inhabitants generally.

